# Phylogeography and population differentiation in *Hepatozoon canis* (Apicomplexa: *Hepatozoidae*) reveal expansion and gene flow in world populations

**DOI:** 10.1186/s13071-021-04924-x

**Published:** 2021-09-14

**Authors:** Antonio Acini Vásquez-Aguilar, Arturo Barbachano-Guerrero, Diego F. Angulo, Víctor Hugo Jarquín-Díaz

**Affiliations:** 1 Red de Biología Evolutiva, Instituto de Ecología, 91073 Xalapa, Veracruz Mexico; 2 Centro de Investigaciones Tropicales, Universidad Veracruzana, Xalapa, Veracruz 91000 México; 3 BioFrontiers Institute, University of Colorado Boulder, Boulder, CO 80303 USA; 4 Experimental and Clinical Research Center, A Cooperation Between the Max-Delbrück-Center for Molecular Medicine in the Helmholtz Association and the Charité-Universitätsmedizin Berlin, Berlin, Germany; 5 Charité-Universitätsmedizin Berlin, Corporate Member of Freie Universität Berlin and Humboldt-Universität Zu Berlin, Experimental and Clinical Research Center, Lindenberger Weg 80, 13125 Berlin, Germany; 6 Max-Delbrück-Center for Molecular Medicine in the Helmholtz Association (MDC), Berlin, Germany

**Keywords:** Dog parasites, Hemoparasites, Tick-borne pathogens, 18S rRNA gene, Ticks

## Abstract

**Background:**

*Hepatozoon canis* is a protozoan transmitted to dogs and other wild carnivores by the ingestion of ticks containing mature oocysts and is considered the principal cause of canine hepatozoonosis in the world. Here, we examined ribosomal RNA 18S gene sequence variation to determine the genetic differences and phylogeographic diversity of *H. canis* from various geographical areas around the world.

**Methods:**

We used 550 publicly available sequences of *H. canis* from 46 countries to assess haplotype relationships, geographical structure, genetic diversity indices, and relationships among populations. We performed neutrality tests and pairwise comparisons of fixation index (*F*_ST_) values between groups and pairwise comparisons of *F*_ST_ values between populations. To determine whether populations are structured, analyses of molecular variance (AMOVAs) and spatial analysis of molecular variance (SAMOVA) were performed.

**Results:**

The dataset of *H. canis* yielded 76 haplotypes. Differentiation among populations indicated that there is no phylogeographical structure (*G*_ST_ = 0.302 ± 0.0475). Moreover, when samples were grouped by continents a significant *F*_ST_ was obtained, meaning that populations were genetically differentiated. The AMOVA showed that 57.4% of the genetic variation was explained by differences within populations when all locations were treated as a single group and revealed that there is no population structure when populations are grouped into two, three, and four groups (*F*_CT_, *p* > 0.05), suggesting that dispersal between populations is high. SAMOVA revealed significant *F*_CT_ values for groups *K* = 5. The Tajima’s *D* and Fu’s *Fs* show that populations have undergone recent expansion, and the mismatch distribution analysis showed population expansion (multimodal distribution).

**Conclusions:**

The current molecular data confirmed that *H. canis* does not show phylogeographic or population structure. The haplotypes exhibit low genetic differentiation, suggesting a recent expansion due to gene flow among populations. These results provide pivotal information required for future detailed population genetic analysis or to establish control strategies of this parasite.

**Graphical abstract:**

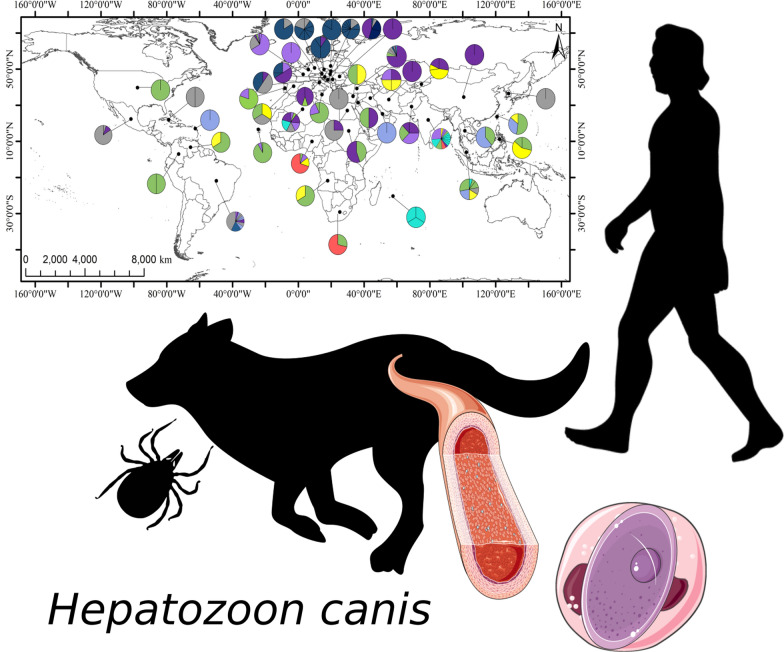

**Supplementary Information:**

The online version contains supplementary material available at 10.1186/s13071-021-04924-x.

## Background

The vector-borne pathogens of the genus *Hepatozoon* infect a wide range of species of mammals, reptiles, amphibians, and birds [1–5]. These parasites are transmitted by different groups of arthropods including ticks, lice, mosquitoes, mites, sand flies, tsetse flies, kissing bugs, and leeches [6–8]. In the past years, the prevalence of *Hepatozoon* species affecting domestic and wildlife animals has increased worldwide; therefore, the study of *Hepatozoon* has gained relevance in the veterinary field [1]. *Hepatozoon americanum* and *H. canis *are protozoa transmitted to dogs and other wild carnivores by ingestion of ticks containing mature oocysts [9, 10]. Although both *H. americanum* and *H. canis* are reported in canids, the latter is widely spread and is considered the principal cause of canine hepatozoonosis in the world [11]. The main vector for *H. canis* is the brown dog tick *Rhipicephalus sanguineus* [9]; however, other tick species are suspected to be possible vectors, for example *Haemaphysalis flava* and *Haemaphysalis longicornis* in Japan [12], *Amblyomma ovale* in Brazil [13], *Amblyomma mixtum* and *Rhipicephalus turanicus* in Mexico [1, 14], and *Ixodes ricinus* ticks in Italy [15]. Following ingestion of infected ticks, *H. canis* sporozoites spread via the bloodstream and lymph to several organs including the spleen, bone marrow, lung, liver, and kidney, infecting leukocytes and parenchymal tissue cells.

Wild canids may be an important reservoir for *H. canis*, thus representing a possible health hazard to domestic dog populations [16]. Wild canids do not develop clinical signs; in contrast, infection in dogs affects several organs resulting in anemia and lethargy [16, 17]. Currently, *H. canis* infects a wide range of carnivorous hosts around the world including dogs, jackals, foxes, opossums, and domestics cats [1, 15, 16, 18–20].

Molecular sequences obtained from collected samples can provide information about past evolutionary events. Relevant probabilistic models of molecular evolution enable reconstructing ancestral sequences to a sample of taxa, and phylogenetics provides an adequate framework for the reconstruction of ancestral sequences [21]. Phylogeographic studies of several taxa have revealed a pattern of lineage divergence associated with contraction to and expansion populations associated with the glacial and interglacial cycles [22] as well as with the formation of geographic barriers [23]. Mutation rates and speed of selection differ among taxa, and in many cases, their dispersion to new areas is mediated by anthropogenic factors such as the mobility of the hosts towards different geographic regions, as has been shown by several studies (i.e. *Vibrio vulnificus* [21], canine distemper virus [24], *Corynosoma australe* [25], *Angiostrongylus cantonensis*, and *A. malaysiensis* [26]) including those performed with some apicomplexans (*Plasmodium knowlesi* [27] and *Toxoplasma gondi* [28]).

Despite the relevance of many parasitic diseases, the studies in genetic and phylogeographic diversity are few compared with other taxa like mammals, amphibians, birds, and plants. Molecular detection of *H. canis* is widely reported in different studies in several regions around the world; however, the genetic diversity and phylogeography of these hemoparasites is understudied [1, 15, 16]. In this article, we examined ribosomal RNA 18S gene sequence variation to determine the genetic differences and phylogeographic diversity of *H. canis* from various geographical areas around the world. For *H. canis*, we can infer that dispersion around the world could be related to the movement of dogs associated with human migration; hence, we hypothesized there is no population or phylogeographic structure. Thus, we aimed to test whether the migratory movements in humans is associated with the population structure of eukaryotic parasites, using *Hepatozoon* as a model of study.

## Methods

### Sequence selection

To define the final dataset of sequences included in this study, several filtering steps were applied as follows: (1) a total of 1170 sequences were downloaded from the ENA/GenBank database using as searching criteria “*Hepatozoon canis*” to discard any sequences not identified to species level (*Hepatozoon* sp.); (2) 55 sequences without complete metadata lacking host and country of origin were discarded; (3) sequences with duplicated accession number (redundant) or missed annotations from parasites different from *H. canis* were not selected; (4) considering an observed wide range in sequence length between 140 bp and 3.1 kbp, sequences < 500 bp were omitted from further analysis. The remaining sequences had a median of 602 bp (533–677). During this step, sequences from genetic regions other than the ribosomal 18S gene were discarded. (5) Finally, the remaining 618 sequences were aligned, and sequences with coverage < 50% (in the final alignment with length of 589 bp) were discarded. The custom R scripts used for the sequence selection are available at: https://github.com/VictorHJD/Hepatozoon_Phylogeography. The script for selection was run in R version 4.0.3 [29].

### DNA sequencing

**Fig. 1:**
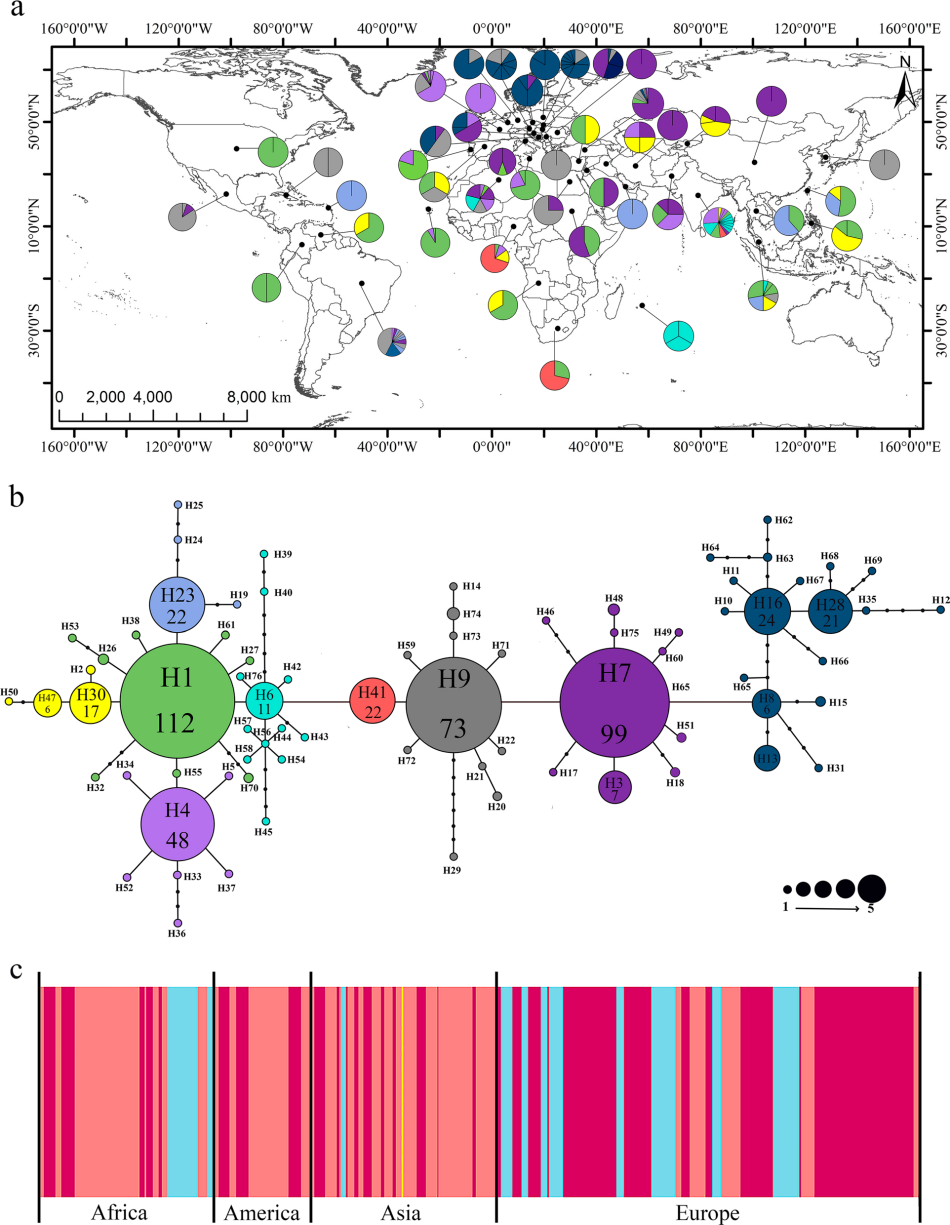
Geographical distribution and statistical parsimony network of 76 rRNA 18S haplotypes found in 46 populations of *H. canis* in the world. **a** Distribution map of haplotypes. Black dots represent sampled populations. Pie charts represent haplotypes found in each sampling population. Section size of pie charts corresponds to the proportion of individuals with a given haplotype. **b** Haplotype network. Black dots represent unsampled haplotypes. Circle size represents the frequency of each haplotype. The coloring facilitates the representation of haplotype diversity in the geography based on the nine most prevalent haplotypes. The numbers inside the haplotypes in the network indicate the number of individuals that share that haplotype. **c** Bayesian analysis of population structure (BAPS). BAPS show four genetic clusters distributed in different proportions among continents. Colors indicate each genetic cluster. The cluster colored in yellow includes individuals from the Asia populations exclusively and hence is less abundant. The cluster in light blue includes individuals from the African, Asian and European populations. The clusters in pink and magenta include individuals from populations in the four continents

The final cured dataset contained 550 sequences (samples) from the rRNA 18S gene spanning the V4 hypervariable region of *H. canis* (Table 1). Sequences belonged to populations from 46 countries (populations) on four continents (Fig. 1, Table 1). These included 109 sequences from Africa, 61 sequences from America, 116 sequences from Asia, and 264 from Europe (Table 1, Additional file 1: Dataset S1). The collected sequences corresponded to isolates from several hosts: dogs (*Canis lupus familiaris*) (*n* = 339), wildcats (*Felis silvestris*) (*n* = 2), red foxes (*Vulpes vulpes*) (*n* = 94), pampas fox, (*Lycalopex gymnocercus*) (*n* = 1), black-backed jackals (*Canis mesomelas*) (*n* = 7), golden jackals (*Canis aureus*) (*n* = 15), opossums (*Didelphis albiventris*) (*n* = 2), and capybara (*Hydrochoerus hydrochaeris*) (*n* = 1) and several tick vector species: *Rhipicephalus sanguineus* (*n* = 46), *Rhipicephalus (Boophilus) microplus* (*n* = 1), *Rhipicephalus tiranicus* (*n* = 7), *Rhipicephalus* spp. (*n* = 3), *Haemaphysalis adleri* (*n* = 2), *Haemaphysalis bispinosa* (*n* = 6), *Haemaphysalis concinna* (*n* = 2), *Haemaphysalis longicornis* (*n* = 1), *Ixodes canisuga* (*n* = 2), *Ixodes hexagonus* (*n* = 1), *Ixodes ricinus* (*n* = 10), *Dermacentor marginatus* (*n* = 2), *Dermacentor reticularis* (*n* = 1), *Dermacentor* spp. (*n* = 1), *Amblyomma mixtum* (*n* = 1), and three unidentified ticks. All sequences were automatically aligned using Clustal X [30] and manually edited with PhyDE-1v0.9971 [31].

**Table 1:** Number of analyzed samples (*n*) for molecular marker (18S rRNA gene) and number of distinct haplotypes (*H*) found in *H. canis* individuals sampled, and the number of individuals per haplotype in parentheses

Pop	Location	Continent	n	Haplotypes
1	Algeria	Africa	19	H3(3), H4(3), **H5(1)**, H6(4), H7(4), H8 (1), H9(3)
2	Angola	Africa	3	H1(2), H2(1)
3	Cape Verde	Africa	45	H1(41), H4(3), H26(1)
4	Egypt	Africa	4	H7(1), H9(3)
5	Mauritius	Africa	3	**H56(1)**, **H57(1)**, **H58(1)**
6	Nigeria	Africa	20	H1(1), H4(2), H30(3), H41(14)
7	South Africa	Africa	7	H41(5), H70(2)
8	Sudan	Africa	8	H1(3), H8(5)
9	Brazil	America	33	H4(1), H7(6), H9(14), **H17(1)**, H18(2), **H19(1)**, H20(2), **H21(1)**, **H22(1)**, H23(2), **H24(1)**, **H25(1)**
10	Colombia	America	2	H1(1), **H27(1)**
11	Cuba	America	2	H9(1), **H29(1)**
12	Mexico	America	19	H7(2), H9(16), **H59(1)**
13	St Kitts	America	1	H23(1)
14	Venezuela	America	3	H1(2), H2(1)
15	USA	America	1	H1(1)
16	China	Asia	1	H7(1)
17	India	Asia	27	H1(2), H4(7), H6(4), H30(1), **H36(1)**, **H37(1)**, **H38(1)**, **H39(1)**, **H40(1)**, H41(3), **H42(1)**, **H43(1)**, **H44(1)**, **H45(1)**, **H46(1)**
18	Iran	Asia	1	H7(1),
19	Iraq	Asia	4	H4(1), H7(1), H30(1), H47(1)
20	Jordan	Asia	2	H1(1), **H49(1)**
21	Kyrgyzstan	Asia	11	H7(1), H47(5), **H50(1)**, H51(2)
22	Malaysia	Asia	18	H1(5), H9(2), H23(4), H26(2), H30(3), **H53(1)**, **H54(1)**
23	Pakistan	Asia	8	H1(2), H4(3), H7(2), **H60(1)**
24	Philippines	Asia	7	H1(2), H30(4), **H61(1)**
25	Qatar	Asia	1	H4(1)
26	South Korea	Asia	2	H9(2)
27	Taiwan	Asia	21	H1(11), H23(7), H30(3)
28	Thailand	Asia	13	H1(5), H23(8)
29	Austria	Europe	10	H9(2), **H10(1)**, **H11(1)**, **H12(1)**, H13(1), **H14(1)**, H15(2), H16(1)
30	Bosnia & H	Europe	10	H3(1), H9(5), H13(4)
31	Croatia	Europe	12	H4(2), H7(6), H16(1), H28(3)
32	Cyprus	Europe	2	H1(1), H30(1)
33	Czech Rep	Europe	10	H7(1), H16(4), H28(4), **H31(1)**
34	France	Europe	32	H3(1), H4(19), H9(7), **H32(1)**, **H33(1)**, **H34(1)**
35	Germany	Europe	6	H9(1), H16(5)
36	Hungary	Europe	31	H7(14), H9(2), H16(6), H28(8), **H35(1)**
37	Italy	Europe	9	H1(4), H7(1), H48(4)
38	Luxembourg	Europe	1	**H52(1)**
39	Malta	Europe	14	H1(10), H4(3), **H55(1)**
40	Poland	Europe	6	H16(5), **H62(1)**
41	Portugal	Europe	16	H1(13), H4(3)
42	Romania	Europe	4	H7(4)
43	Serbia	Europe	9	H9(9)
44	Slovakia	Europe	19	H9(3), H16(2), H28(6), H63(2), **H64(1)**, **H65(1)**, **H66(1)**, **H67(1)**, **H68(1)**, **H69 (1)**
45	Spain	Europe	3	H1(1), H39(1), **H71(1)**
46	Turkey	Europe	70	H1(4), H6(3), H7(52), H9(3), **H72(1)**, **H73(1)**, H74(4), **H75(1)**, H76**(1)**

Codes are from networks in Fig. 1. Exclusives haplotypes for each population are shown in bold letters

### Haplotype relationships

To infer genealogical relationships among *H. canis* haplotypes, statistical parsimony networks (haplotypes network) were constructed using the program TCS 1.2.1 [32]. The aligned sequences were mapped to estimate gene genealogies with gaps treated as missing data and a 95% connection probability limit. Loops were resolved following the criteria given by Pfenninger and Posada [33]. The full dataset was processed blindly and treated independently from their host of origin.

The most likely number of genetically differentiated clusters was estimated using BAPS (Bayesian Analysis of Population Structure, version 6.0) [34]. BAPS assesses the most likely number of genetically different clusters using the module for linked molecular data. The codon linkage model, appropriate for our sequence data, was applied. For the chosen marker, the probability of a different number of genetic clusters (*K* = 2 to *K* = 10) under the independent loci model was evaluated. Two independent runs with ten replicates for each *K*, accepting the partition with the *K *value that had the highest likelihood and posterior probability (PP) provided the final result.

### Geographical structure, genetic diversity indices, and relationships among populations

Population diversity indices for unordered (*h*_S_, *h*_T_) and ordered haplotypes (*v*_S_, *v*_T_) and differentiation parameters (*G*_ST_, *N*_ST_) were estimated using software PERMUT 1.0 [35]. The within-population diversity (*h*_S_), total diversity (*h*_T_), geographical average haplotype diversity (*v*_S_), geographical total haplotype diversity (*v*_T_), level of population differentiation at the species level (*G*_ST_), and an estimate of population subdivisions for phylogenetically ordered alleles (*N*_ST_) were calculated. *G*_ST_ and *N*_ST_ are often used to assess the geographical structure affecting population differentiation. Significant differences between *N*_ST_ and *G*_ST_ parameters were tested with 10,000 permutations. If *N*_ST_ is significantly larger than *G*_ST_, this means that two alleles sampled from the same region are phylogenetically more closely related than two alleles sampled from different regions, evidencing the presence of a significant phylogeographical signal in the data [36].

Neutrality tests and pairwise comparisons of *F*_ST_ values between continents were calculated using ARLEQUIN 3.11 [36]. Pairwise comparisons of *F*_ST_ values between populations were calculated with 1000 permutations. Populations with three samples or fewer were excluded as required for this analysis.

To determine whether populations were structured, analyses of molecular variance (AMOVAs) [37] were performed based on pairwise differences. The AMOVA was carried out considering all the populations across the sampling area as a single unit. Furthermore, hierarchical AMOVA was performed with populations treated as (i) grouped into two, New world and Old world (America, Europe + Africa + Asia), or (ii) three (America, Africa, Asia + Europe), according to the hypothesis that *H. canis* arises in Eurasia and spreads to Africa and America, or (iii) four groups (America, Africa, Asia, Europe) similar to the episode where the four geographic regions (continents) have been isolated long enough to differentiate populations. The AMOVAs were performed using ARLEQUIN 3.11 with 16,000 permutations to determine the significance of each AMOVA. Additionally, spatial analysis of molecular variance (SAMOVA) was performed using SAMOVA 1.0 [38] to identify clusters of populations that are geographically homogeneous and genetically differentiated.

### Demographic history

Molecular diversity indices, including the number of haplotypes (*N*_H_), gene diversity (*h*), nucleotide diversity ($\pi$), and pairwise comparisons of *F*_ST_ values between populations, were calculated using ARLEQUIN 3.11 [36]. We used mismatch distributions to assess population expansion and time variation in effective population size (Ne). The historical population expansions were analyzed with Tajima’s *D* (*D*_T_) [39] and Fu’s (*F*_S_) [40]. Negative values in *D*_T_ and *F*_S_ that result from an excess of rare alleles present at low frequencies indicate that populations have undergone recent expansion.

Neutrality tests were performed in ARLEQUIN 3.11, with 10,000 permutations [36]. Mismatch distributions [41] were calculated using the sudden expansion model of Schneider & Excoffier [42], with 1000 bootstrap replicates. Mismatch distributions [43] and Harpending’s raggedness index (Hri; [41]) were calculated in DnaSP v.6 [44]. The validity of the sudden expansion assumption was determined using the sum of squares differences (SSDs) and Hri [41], both of which are higher in stable, non-expanding populations [43]. Low and non-significant values of Hri and SDD indicated a good fit between the observed and the expected values of the sudden expansion model.

Ramos-Onsins and Rozas *R*_*2*_ statistics were calculated based on the difference between the number of singleton mutations and the average number of nucleotide differences, where small positive values of *R*_*2*_ are expected under a scenario of population expansion [45]. Significance was evaluated by comparing observed values with null distributions generated by 10,000 replicates, using the empirical population sample size and the observed number of segregating sites in the “pegas” package of R version 4.0.3 [29].

Finally, the Bayesian skyline plot [46] method implemented in BEAST 2.6.4 [47] was performed to further explore the demographic history and to estimate the timing of the population expansion in *H. canis* populations (four continents and all continents). The 550 sequences were used to run the analysis using the coalescent Bayesian skyline tree prior. This coalescent-based approach estimates the posterior distribution of effective population size (Ne) at intervals along a phylogeny, thereby allowing one to infer population fluctuations over time. The haplotype sequence alignments were imported in the program BEAUti2 (included in BEAST 2.6.4) in nexus format. The best nucleotide substitution model was estimated using Jmodeltest 2.0 [48]. The HKY + G substitution model was the best model identified for African, American and European *H. canis*, while that for Asia was TPM3uf. Finally, when populations were treated as a single group, the best substitution model was TPM3uf + G. Since the literature lacks estimations of substitution rates for *H. canis,* a substitution rate of 1.5% per 100 million years for the 18S rRNA used to study the lineage evolution of *Toxoplasma gondii* [49] was implemented in the analysis. Three independent runs of 10 million generations each were run, with trees and parameters sampled every 1000 iterations, with a burn-in of 10%. Results of each run were visualized using TRACER to ensure that stationarity and convergence had been reached [50].

## Results

### A highly abundant haplotype of *H. canis* is widely spread worldwide

Statistical parsimony retrieved a well-resolved haplotype network that identified 76 haplotypes in the world representing four haplogroups (Fig. 1, Additional file 2: Figure S1). The aligned 18S rRNA gene dataset for 550 samples of *H. canis* (mean length: 586 bp) yielded 76 haplotypes with different abundance along the geography (Table 1, Fig. 1a). The haplotype H1 (20.3%, 112/550 sequences) was the most common and widespread of the individuals and in 43.4% (20/46 total populations, considering each set of sequences from the same country as a different population), retrieved in populations located in the four continents. It was followed by haplotypes H7 (18%, 99/550 sequences) in populations located in the Old World and H9 (13.3%, 73/550 sequences) retrieved in populations located in the four continents. Haplotypes H13, H24, and H16 were exclusively found in Europe, and haplotype H41 was only retrieved in Africa and Asia (Fig. 1). The haplotypes did not cluster based on their host of origin, suggesting the lack of variability of the sequences between invertebrate and vertebrate hosts (Additional file 2: Table S1, Figure S2).

Out of total sequences in H1, 13.4% (15/112) correspond to tick while 84.8% (95/112) belong to dogs and two sequences (1.7%) to red foxes. In H7, 9.09% (9/99) belong to ticks, and the 90.91% are distributed in sequences from other vertebrate hosts (74/99; 74.74% belonged to dogs, 10/99; 10.1% to red foxes, and 6/99; 6.06% to golden jackals). For H9, 12.32% (9/73) belong to ticks, 53.42% (39/73) to dogs, 30.13% (22/73) to red foxes, 2.73% (2/73) to opossum, and 1.38% (1/73) to pampas fox. Moreover, the haplotype network shows various singletons for each host (Additional file 2: Figure S2).

BAPS analyses with sequences indicated the existence of four genetic clusters (*K* = *4*) (log marginal likelihood =  − 4667.6928, PP = 1.0) (Fig. 1c). The four genetic clusters from the BAPS analysis distributed in different proportions among continents. The yellow cluster includes individuals from the Asian populations, exclusively. The light blue cluster gathers individuals from the African, Asia and European populations exclusively. The pink and magenta clusters include individuals from populations distributed in the four continents, confirming the lack of genetic structure (Fig. 1c). BAPS analysis was in agreement with the shallow genetic divergence and haplotype distribution shown in Fig. 1b.

### Lack of phylogeographic structure of *H. canis* suggests gene flow between populations

Differentiation among populations based on 18S rRNA gene variation (mean ± SE, *G*_*S*T_ = 0.302 ± 0.0475) indicated that *H. canis* is genetically subdivided. Genetic diversity across all populations (*h*_T_ = 0.913 ± 0.0136; *v*_T_ = 0.914 ± 0.0616) was higher than the average within-population value (*h*_S_ = 0.637 ± 0.0463; *v*_S_ = 0.550 ± 0.0682), indicating that populations are similar among them. PERMUT analysis showed that *N*_ST_ was not significantly different (PERMUT: *N*_ST_ = 0.399 ± 0.0578 vs. *G*_ST_ = 0.302 ± 0.0475, perm: 10,000, *p* > 0.05) than *G*_ST_, indicating no phylogeographical structuring.

*Hepatozoon canis* is genetically differentiated between populations, as pairwise comparisons of *F*_ST_ values were significant for the data set when samples were grouped by continents (Table 2). Europe was the continent that most differed from the other continents. The lowest differentiation was observed between Asia and Africa, while the highest difference was observed between Asia and Europe (Table 2).

**Table 2:** Genetic pairwise comparisons of *F*_ST_ values for 18S rRNA among groups of *H. canis* populations

Group	Africa	America	Asia	Europa
Africa	–			
America	0.2500	–		
Asia	0.0293	0.2082	–	
Europe	0.2178	0.1054	0.32127	–

All values had significant values at *p* < 0.001

Each continent is consistent with the four haplogroups estimated by haplotype network

The AMOVA results showed that 57.4% of the genetic variation was explained by differences within populations when all locations were treated as a single group (Table 3). The hierarchical AMOVA revealed that there is no population structure, with the lowest *F*_CT_ value obtained when populations are grouped into two, three, and four groups with differences that were not statistically significant (Table 3), suggesting that dispersal of *H. canis* between populations is high, thus increasing gene flow. SAMOVA results revealed significant *F*_CT_ values for groups between *K* = *5*. The highest *F*_*C*T_ value was for *K* = *5* (Table 3). When *K* = *6*, FCT was smaller than the *F*_CT_ of *K* = *5*, and an additional increase in the number of *K* led to a dissolution of group structure, and single-population groups were formed.

**Table 3:** Results of AMOVA models on *H. canis* populations with no groups defined a priori (a) and (b) grouped into two groups, New World and Old World (America, Europe + Africa + Asia) and (c) grouped into three groups (America, Africa, Asia + Europe) or (d) four groups (America, Africa, Asia, Europe) according to geographical distribution in the continents

	d*f*	Sum of squares	18S rRNA	%	Fixation indices
			Estimated variance		
a. No groups defined					
Among populations	35	604.762	1.0673	42.62	
Within populations	214	734.886	1.4297	57.38	*F*_ST_ = 0.42***
Total	549	1339.647	2.4818		
b. Two groups					
Among groups	1	31.568	0.0315	1.25	*F*_CT_ = 0.01ns
Among pop. within groups	34	573.194	1.0555	41.94	*F*_SC_ = 0.42***
Within populations	514	734.886	1.4297	56.81	*F*_ST_ = 0.43 ***
Total	549	1339.647	2.5168		
c. Three groups					
Among groups	2	78.347	0.0844	3.33	*F*_CT_ = 0.03ns
Among pop. within groups	33	526.414	1.0200	40.25	*F*_SC_ = 0.41***
Within populations	514	734.886	1.4297	56.42	*F*_ST_ = 0.43***
Total	549	1339.647	2.5342		
d. Four groups					
Among groups	3	178.689	0.2979	11.56	*F*_CT_ = 0.11ns
Among pop. within groups	32	426.072	0.8504	32.99	*F*_SC_ = 0.37***
Within populations	514	734.886	1.4297	76.02	*F*_ST_ = 0.44***
Total	549	1339.647	2.5781		
e. SAMOVA					
Among groups	4	438.802	1.2680	11.56	*F*_CT_ = 0.42***
Among pop. within groups	31	165.959	0.2722	32.99	*F*_SC_ = 0.15***
Within populations	514	734.886	1.4297	76.02	*F*_ST_ = 0.51***
Total	549	1339.647	2.9699		

*ns* not significant (*p* > 0.05), **p < 001, ***p < 0.0001

SAMOVA *K* = *5* groups

### *Hepatozoon canis* populations have a recent demographic history

**Fig. 2:**
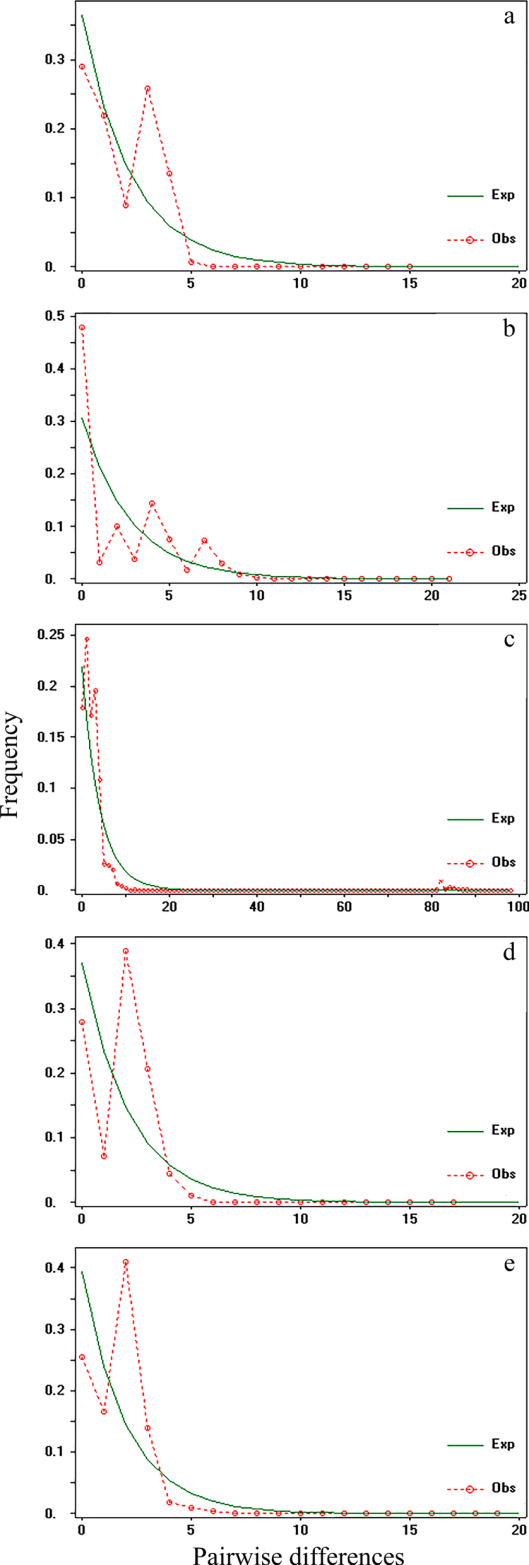
Mismatch distributions with sudden demographic/population expansion model. **a** Expansion model of *Hepatozoon canis* Africa population. **b** Expansion model of *Hepatozoon canis* America population. **c** Expansion model of *Hepatozoon canis* Asia population. **d** Expansion model of *Hepatozoon canis* Europe population. **e** Expansion model of *Hepatozoon canis* global population. Red dotted lines show the observed frequency, and green continuous lines show the expected frequency

Considering the hypothesis of enough divergence time to form independent populations by continent, the Tajima’s *D* and Fu’s *Fs* show a small, statistically significant, negative value for America (Tajima's *D* test: *D* = − 1.5908, SD = 0.9046, *p* = 0.0288; Fu’s Fs test: Fs = − 4.0733, SD = 0.18721,* p* = 0.0288), Asia (Tajima’s *D* test: *D* = − 2.7120, SD = 1.0353, *p* = 0.0000; Fu’s F*s* test: Fs = − 5.2295, SD = 0.18721,* p* = 0.08600), and Europe (Tajima’s *D* test: *D* = − 1.6212, SD = 1.0353, *p* = 0.0240; Fu’s *Fs* test: Fs = − 13.3581, SD = 5.9440,* p* = 0.0020), and a small, statistically non-significant, negative value for Africa (Tajima’s *D* test: *D* = − 0.1857, SD = 1.0353,* p* = 0.5100; Fu’s Fs test: Fs = − 1.1153, SD = 5.9440,* p* = 0.4230). When populations are treated as a single group, the Tajima’s *D* and Fu’s *Fs* had a small and statistically significant negative value (Tajima’s *D* test: *D* = − 2.5002, SD = 0.0000,* p* = 0.0000; Fu’s Fs test: Fs = − 24.7563, SD = 0.0000,* p* = 0.0000), indicating that populations have undergone recent expansion, often preceded by a bottleneck (Table 4). In the mismatch distribution, SSD and Hri results were low and non-significant (Mismatch analysis test: SSD = 0.0146, SD = 0.0000,* p* = 0.0500; Hri = 0.0348, SD = 0.0000,* p* = 0.0900), considering all populations as one group (global) and grouping America (mismatch analysis test: SSD = 0.0853, SD = 0.0427, *p* = 0.1900; Hri = 0.2013, SD = 0.0963,* p* = 0.1500), Asia (mismatch analysis test: SSD = 0.0145, SD = 0.0427, *p* = 0.1100; Hri = 0.0589, SD = 0.0963,* p* = 0.0600), and Europe populations Asia (mismatch analysis test: SSD = 0.0154, SD = 0.0427, *p* = 0.4100; Hri = 0.3628, SD = 0.0963,* p* = 0.2900), providing evidence for sudden demographic expansion in the past for all groups of populations (Table 4). The mismatch distribution analysis showed a multimodal distribution when observed frequencies were compared against expected frequencies, implying population expansion (Fig. 2). Finally, *R*_2_ had small, statistically significant, positive values for Africa, America, Asia, Europa, and the global populations, indicating recent demographic expansion (Table 4, Fig. 2).

**Table 4:** Summary statistics of demographic analysis of *H. canis* samples in four groups (America, Africa, Asia, Europe) resembling geographical history to infer demographic range expansion

Parameter	Africa	America	Asia	Europe	Global
18S					
*N*	109	61	116	264	550
*N*_H_	15	21	27	37	76
*h*	0.7698 ± 0.0349	0.7246 ± 0.0586	0.8889 ± 0.0161	0.8588 ± 0.0132	0.8937 ± 0.006
*π*	0.0068 ± 0.0038	0.0060 ± 0.0034	0.0100 ± 0.0054	0.0069 ± 0.0038	0.008668 ± 0.004681
*D*_T_	− 0.1857	− 1.5908*	− 2.7120***	− 1.6212*	− 2.5002***
*F*_S_	− 1.1153	− 4.0733*	− 5.2295	− 13.3581***	− 24.7563 ***
SSD	0.0924*	**0.0853**	**0.0145**	**0.0154**	**0.0146**
Hri	0.2244***	**0.2013**	**0.0589**	**0.0362**	**0. 0348**
*R*_*2*_	0.0044***	0.0388**	0.0352*	0.0033***	0.0123**

*N*, number of individuals; *N*_H_, number of haplotypes; *h*, gene diversity; *π*, nucleotide diversity; *D*_T_, Tajima’s *D*; *F*_S_, Fu’s Fs; SDD, differences in the sum of squares or mismatch distribution; Hri, Harpending’s raggedness index

*p < 0.05; **p < 0.01; ***p < 0.001. *D*_T_ and *F*_*S*_ positive values are indicative of mutation-drift-equilibrium, which is typical of stable populations, and negative values that result from an excess of rare haplotypes indicate that populations have undergone recent expansions, often preceded by a bottleneck. Significantly negative values (at the 0.05 level) in both tests reveal historic demographic expansion events. Significant (*p* ≤ 0.05) SSD and Hri values indicate deviations from the sudden expansion model. Values that are consistent with demographic expansion are shown in bold. *R*_2_, Ramos-Onsins and Rozas statistic, small positive values of *R*_2_ are expected under a scenario of population expansion

**Fig. 3:**
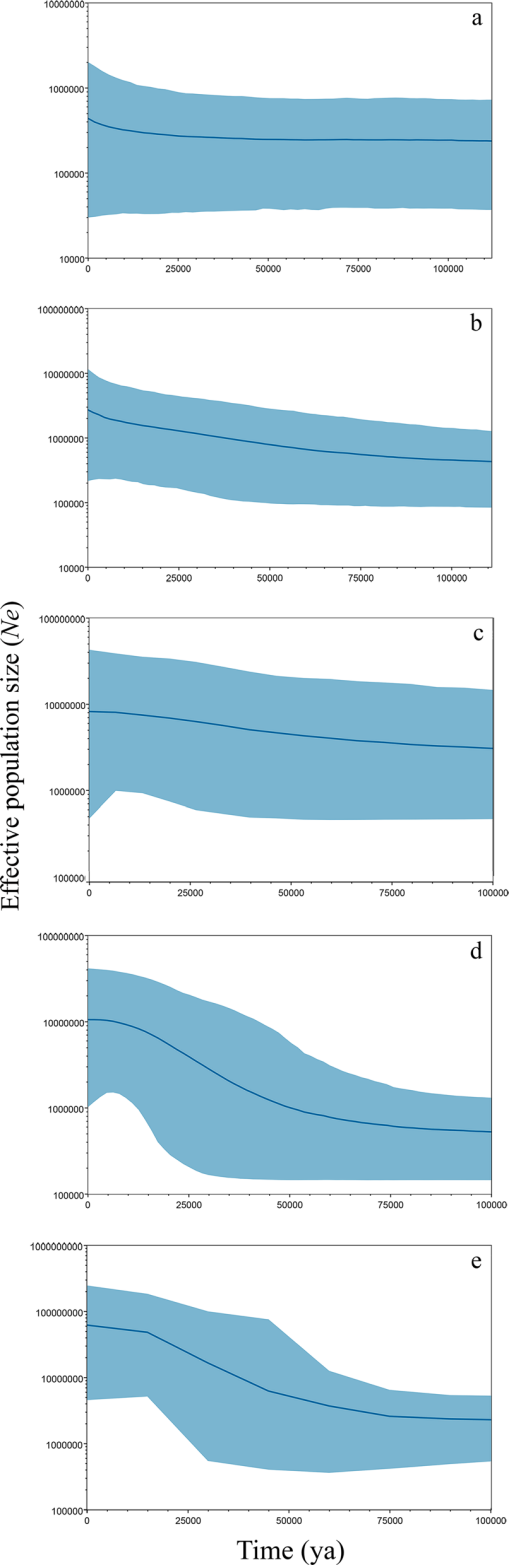
Bayesian skyline plots showing historical demographic trends of *H. canis* in the world. The x axis represents time in years and the y axis is the effective population size (Ne); the light blue area around the blue line shows the 95% highest posterior density (HPD) limits. **a** Variation in the effective population size (Ne) of *Hepatozoon canis* Africa population. **b** Ne of *Hepatozoon canis* America population. **c** *Hepatozoon canis* Asia population. **d** Ne of *Hepatozoon canis* Europe population. **e** Ne of *Hepatozoon canis* global population

The Bayesian skyline plots suggest that population size effectively increased around 75,000–5000 years ago in all populations (Fig. 3). The African population was relatively stable over time, showing a slight increase around 5000 years ago. In contrast, the American population and Asia population increased around 75,000–5000 years ago. The Bayesian skyline plots from the European population and World population indicated a marked *Ne* increase around 25,000–5000 years ago, consistent with a more recent and faster population expansion (Fig. 3d, e).

## Discussion

Our results showed no structured genetic patterns in *H. canis* populations, suggesting high levels of gene flow. Likewise, our results provide evidence of past and recent population expansions that could be due to human migrations carrying their cats and dogs as pets. These results are discussed in detail below.

Since its first description in 1905 [51], *H. canis* has been reported in several countries of America, Europe, Asia, Africa, and Australia, [1, 15, 52–54]. Most studies lack genetic information [54], reporting the presence of *H. canis* by histopathological or microscopic diagnosis [55], which complicates the better understanding of the genetic structure and gene flow of their populations. Our survey of 18S rRNA gene sequence data in populations of *H. canis* identified four haplogroups in the world (Fig. 1). However, haplogroup structure was not geographically congruent nor did it fit previous hypotheses proposed in the past [25, 26]. The more frequently found haplotypes (H1, H4, H7, H9, H16, H23, H28, and H41) are distributed in multiple populations in the four continents, and they come from different hosts. This differs from other studies performed with parasites like *Corynosoma australe* [25] (cytochrome *c* oxidase subunit I (*cox 1*) sequences), *Angiostrongylus cantonensis* and *A. malaysiensis* [26] (partial 66-kDa protein gene sequence), or *Plasmodium knowlesi* [27] (*cox-1* and 18S RNA sequences), which showed haplogroups defined in the network and with geographic congruence. However, these studies used DNA fragments shorter than the data used in our present study. On the other hand, our results agreed with a previous study that reported that dogs carried the same or very similar *H. canis* haplotypes as red foxes, suggesting that both hosts participate in the same epidemiological cycle [56]. In contrast, in this study, the similarities in the haplotypes between host species suggest cross-species transmission, which could be due to (i) the mobility of diverse tick species and their host across the world and (ii) shared habitats of the different host species, both promoting greater gene flow between populations (Fig. 1).

Haplotype diversity (*h*), a measure of species evenness, was high for all populations (Table 4), which may indicate a sustained transmission of *H. canis* in the world over long time periods and high gene flow. Similar high haplotype diversity values have been reported for other parasites such as *P. knowlesi* [27], widely distributed in Southeast Asia. Nucleotide diversity was low in our study, irrespective of the samples geographic or host origins, suggesting that only minor differences occurred between the haplotypes observed, as has been suggested for other protozoa [57, 58]. The haplotype diversity and distribution could be related to multiples factors associated with *H. canis* life cycle, transmission cycle, and dispersion capacity. Our results showed that genetic diversity across all populations was higher than within-populations and genetic differentiation analyses indicated no phylogeographical structuring. These results could be due to the existence of strong gene flow between populations as shown in other species (*Cryptosporidium*, *Giardia*, *Fasciola*) [59, 60]. Inferred patterns of genetic differentiation in *H. canis* do not appear to be correlated with the geographical regions (Fig. 1) and could be associated to diverse human migration events (travel and trade) as reported with other dog pathogens [24, 60].

*F*_ST_ pairwise comparisons, AMOVAs, and BAPS (Tables 2, 3, Fig. 1C) suggest low levels of genetic structuring and the existence of four genetic groups. Altogether these results suggest that dispersal of *H. canis* between populations is high. This pattern is commonly reported for other taxa (e.g. *Psittacanthus calyculatus*,* canine distemper virus*, *Corynosoma austral*, *Fasciola hepatica*) [24–26, 59]. Our results showed low structure and high genetic flow among domestic and wildlife hosts, and among populations worldwide. However, more studies are required to predict the most probable reservoirs for this parasite.

Human activities have affected the geographical ranges of many host species, shrinking by loss of habitat or increasing through introduction of individuals into new regions and promoted spillover events. In parasitic nematodes, the impact of human activity is well documented, offering an opportunity to study how changes in host population size and connectivity shape the population genetics structure [61]. In this study, we infer that the geographical structure, genetic diversity, and relationships among populations in *H. canis* are affected by anthropogenic activities.

Our results showed that *H. canis* populations had a recent expansion (Table 4, Fig. 3). A signature of population expansion was also evident from the multi modal shape of the pairwise mismatch distribution of the *18S rRNA* genes (Fig. 2) and Bayesian skyline plots (Fig. 3). Despite our results confirming the hypothesis of population expansion, these findings cannot define the geographical origin of this expansion. A further analysis dating the evolutionary divergence within Apicomplexa phylum could provide a refined overview regarding the origin of *H. canis*. Historical demographic expansions were determined by analyzing the frequency distributions of pairwise differences between sequences [36, 40]. Neutrality tests with Tajima’s *D* and Fu’s Fs statistics estimate the deviation from neutrality, based on the expectation of a constant population size at mutation-drift equilibrium. Here, a negative Tajima’s *D* signifies an excess of low frequency polymorphisms relative to expectation, indicating population size expansion or positive selection [62]. *Hepatozoon canis* populations displayed a genetic pattern typical of a population that has undergone a recent expansion, and the range of expansion could be a recent phenomenon (5000 years ago), at least that shows the Bayesian skyline plots analysis for African population and when all population were analyzed like a group (Fig. 3). These populations may not have achieved the migration-drift equilibrium, as shown by the lack of phylogeographical structure [39, 40, 62]. The discrepancy in detecting population expansion, based on the Tajima’s *D*, Fu’s *F*_S_, and *R*_2_ statistics, reflects different responses to past changes (e.g. population reduction, population subdivision, a recent bottleneck) or high gene flow between populations [63]. However, these changes could be recent in evolutionary times and could be related to anthropogenic factors, as reported in other studies carried out with canid pathogens [24]. Moreover, the ability of *H. canis* to spillover among host species is feasible given the wide range of carnivores that can be infected by these hemoparasites worldwide [1, 16, 18–20].

In the mismatch distribution result, *H. canis* presented a multimodal pattern suggesting recent expansion. Furthermore, non-significant values for *SSD* mean that the observed data do not deviate from that expected under the model of expansion, and the non-significant raggedness index also indicates population expansion. These results suggest that population expansion occurred recently, except to Africa [43]. Demographic expansion supported by the statistics of demographic analysis and the mismatch distribution as well as the pattern shown by the haplotype network reflects the existence of many different and rare haplotypes (high value of *h*, Table 4, Fig. 1), possibly because not enough time elapsed to accumulate genetic differences. Metazoonosis, like hepatozoonosis, involves complex interactions among the hosts, parasite, and environmental factors [64]. Owing to the historical relationship between dogs and humans (since domestication in Siberia ∼ 23,000 years ago) [65], high mobility from the *H. canis* host favors dispersion and gene flow among populations. The latter is supported by several studies done with dogs and wild canids worldwide, which observed the interactions networks among parasites, vectors, and hosts [1, 20, 52, 54].

The detection of *H. canis* in felids and capybaras highlights the lack of information on the transmission dynamics of *H. canis* into new hosts [6, 8, 66]. Moreover, it shows how disregarded the role of other little-studied tick species, such as *A. mixum*, as potential vectors has been [1]. Although these isolates in non-canid hosts represent incidental findings, this supports the hypothesis that *H. canis* moves between different host geographic areas and vectors favoring gene flow.

## Conclusion

This study provides novel and significant insights into the phylogeography and population differentiation of *H. canis* in the world. The current molecular data confirmed that *H. canis* does not show phylogeographic and population structure, which could be due to its wide range of definitive host (domestics and wild canids), lack of clear understanding of the role of other non-canid species in the transmission dynamics, and the impact of human activities. Our results showed identical *H. canis* haplotypes co-occurring in several geographical regions and host species, indicating wide distribution of the parasite. The haplotypes exhibit low genetic differentiation, suggesting a recent expansion due to gene flow among populations. The reasons are mainly still unknown. However, we consider that the role of human migrations might cause the dispersion of vector and host. Finally, using more variable target regions such as mitochondrial and apicoplast genomes might improve resolution. We suggest these more variable regions should be applied in future studies to understand the natural history of *H. canis* and approach it from an eco-epidemiological vision.

## Supplementary Information

**Additional file 1: Dataset S1.** GenBank accession numbers for the sequences used on this study.

**Additional file 2: Figure S1.** Statistical parsimony network of 76 rRNA 18S haplotypes found in 46 populations of *H. canis* worldwide. Black dots represent unsampled haplotypes. The frequency of each haplotype is represented by the size of the circle. Each haplotype is coded with a different color according to the continent. The numbers inside the haplotypes in the network indicate the number of individuals that share that haplotype. The four rectangles represent the four haplogroups reported in this study. **Figure S2.** Geographical distribution of host and statistical parsimony network of 76 rRNA 18S haplotypes found of *H. canis* in the world. *a* Distribution map of the hosts. Black dots represent sampled populations and pie charts represent hosts found in each sampling population. Section size of pie charts corresponds to the proportion of individuals with given hosts. *b* Haplotype network. Black dots represent unsampled haplotypes. The frequency of each haplotype is represented by the size of the circle. Each haplotype is coded with a different color according to the hosts. The numbers inside the haplotypes in the network indicate the number of individuals that share that haplotype. **Table S1.** Number of analyzed samples (n) for molecular marker (18S rRNA gene) and hosts found of *H. canis.*

## Data Availability

Data supporting the conclusions of this article are included within the article and its additional files.
