# Supplementary material for: Phylogeography and population differentiation in Hepatozoon canis (Apicomplexa: Hepatozoidae) reveal expansion and gene flow in world populations
Source: Parasit Vectors. 2021 Sep 14;14:467. doi: 10.1186/s13071-021-04924-x (PMC8439048; doi:10.1186/s13071-021-04924-x)
Supplement: Supplementary file 2 — Additional file 2: Figure S1. Statistical parsimony network of 76 rRNA 18S haplotypes found in 46 populations of H. canis worldwide. Black dots represent unsampled haplotypes. The frequency of each haplotype is represented by the size of the circle. Each haplotype is coded with a different color according to the continent. The numbers inside the haplotypes in the network indicate the number of individuals that share that haplotype. The four rectangles represent the four haplogroups reported in this study. Figure S2. Geographical distribution of host and statistical parsimony network of 76 rRNA 18S haplotypes found of H. canis in the world. a Distribution map of the hosts. Black dots represent sampled populations and pie charts represent hosts found in each sampling population. Section size of pie charts corresponds to the proportion of individuals with given hosts. b Haplotype network. Black dots represent unsampled haplotypes. The frequency of each haplotype is represented by the size of the circle. Each haplotype is coded with a different color according to the hosts. The numbers inside the haplotypes in the network indicate the number of individuals that share that haplotype. Table S1. Number of analyzed samples (n) for molecular marker (18S rRNA gene) and hosts found of H. canis. [file 13071_2021_4924_MOESM2_ESM.docx]

**Phylogeography and population differentiation in *Hepatozoon canis* (Apicomplexa: *Hepatozoidae*) reveal expansion and gene flow in world populations**

Antonio Acini Vásquez-Aguilar, Arturo Barbachano-Guerrero, Diego F. Angulo & Víctor Hugo Jarquín-Díaz.

Additional file 2:

**Table S1. Number of analysed samples (n) for molecular marker (18S rRNA gene) and hosts found of *H. canis***

| **Pop.** | **Location** | **Continent** | **n** | **Hosts** |
| --- | --- | --- | --- | --- |
| 1 | Algeria | Africa | 19 | Dogs (19) |
| 2 | Angola | Africa | 3 | Dogs (3) |
| 3 | Cape Verde | Africa | 45 | Dogs (45) |
| 4 | Egypt | Africa | 4 | Ticks (4) |
| 5 | Mauritius | Africa | 3 | Dogs (3) |
| 6 | Nigeria | Africa | 20 | Dogs (10), Ticks (10) |
| 7 | South Africa | Africa | 7 | Black-Backed jackals (7) |
| 8 | Sudan | Africa | 8 | Dogs (8) |
| 9 | Brazil | America | 33 | Dogs (22), Ticks (5), Wildcats (2), Opossums (2), Pampas fox (1), Capybara (1) |
| 10 | Colombia | America | 2 | Dogs (2) |
| 11 | Cuba | America | 2 | Dogs (2) |
| 12 | Mexico | America | 19 | Dogs (18), Ticks (1) |
| 13 | St Kitts | America | 1 | Dogs (1) |
| 14 | Venezuela | America | 3 | Dogs (3) |
| 15 | USA | America | 1 | Dogs (1), |
| 16 | China | Asia | 1 | Ticks (1) |
| 17 | India | Asia | 27 | Dogs (13), Ticks (14) |
| 18 | Iran | Asia | 1 | Dogs (1) |
| 19 | Iraq | Asia | 4 | Dogs (1), Red foxes (2), Golden jackal (1) |
| 20 | Jordan | Asia | 2 | Dogs (2) |
| 21 | Kyrgyzstan | Asia | 11 | Dogs (11) |
| 22 | Malaysia | Asia | 18 | Dogs (17), Ticks (1) |
| 23 | Pakistan | Asia | 8 | Dogs (8) |
| 24 | Philippines | Asia | 7 | Dogs (7) |
| 25 | Qatar | Asia | 1 | Dogs (1) |
| 26 | South Korea | Asia | 2 | Dogs (2) |
| 27 | Taiwan | Asia | 21 | Dogs (2), Ticks (19) |
| 28 | Thailand | Asia | 13 | Dogs (13) |
| 29 | Austria | Europe | 10 | Red foxes (3), Golden jackal (7) |
| 30 | Bosnia & H. | Europe | 10 | Dogs (7), Red foxes (3) |
| 31 | Croatia | Europe | 12 | Dogs (10), Red foxes (2) |
| 32 | Cyprus | Europe | 2 | Dogs (2) |
| 33 | Czech Rep. | Europe | 10 | Dogs (9), Ticks (1) |
| 34 | France | Europe | 32 | Red foxes (32) |
| 35 | Germany | Europe | 6 | Red foxes (1), Ticks (5) |
| 36 | Hungary | Europe | 31 | Dogs (10), Red foxes (11), Golden jackal (6), Ticks (4) |
| 37 | Italy | Europe | 9 | Dogs (1), Red foxes (6), Ticks (2) |
| 38 | Luxemburgue | Europe | 1 | Ticks (1) |
| 39 | Malta | Europe | 14 | Dogs (14) |
| 40 | Poland | Europe | 6 | Red foxes (6) |
| 41 | Portugal | Europe | 16 | Dogs (15), Ticks (1) |
| 42 | Romania | Europe | 4 | Red foxes (4) |
| 43 | Serbia | Europe | 9 | Dogs (1), Red foxes (8) |
| 44 | Slovakya | Europe | 19 | Dogs (1), Red foxes (13), Ticks (5) |
| 45 | Spain | Europe | 3 | Red foxes (1), Ticks (2) |
| 46 | Turkey | Europe | 70 | Dogs (54), Red foxes (3), Ticks (13) |


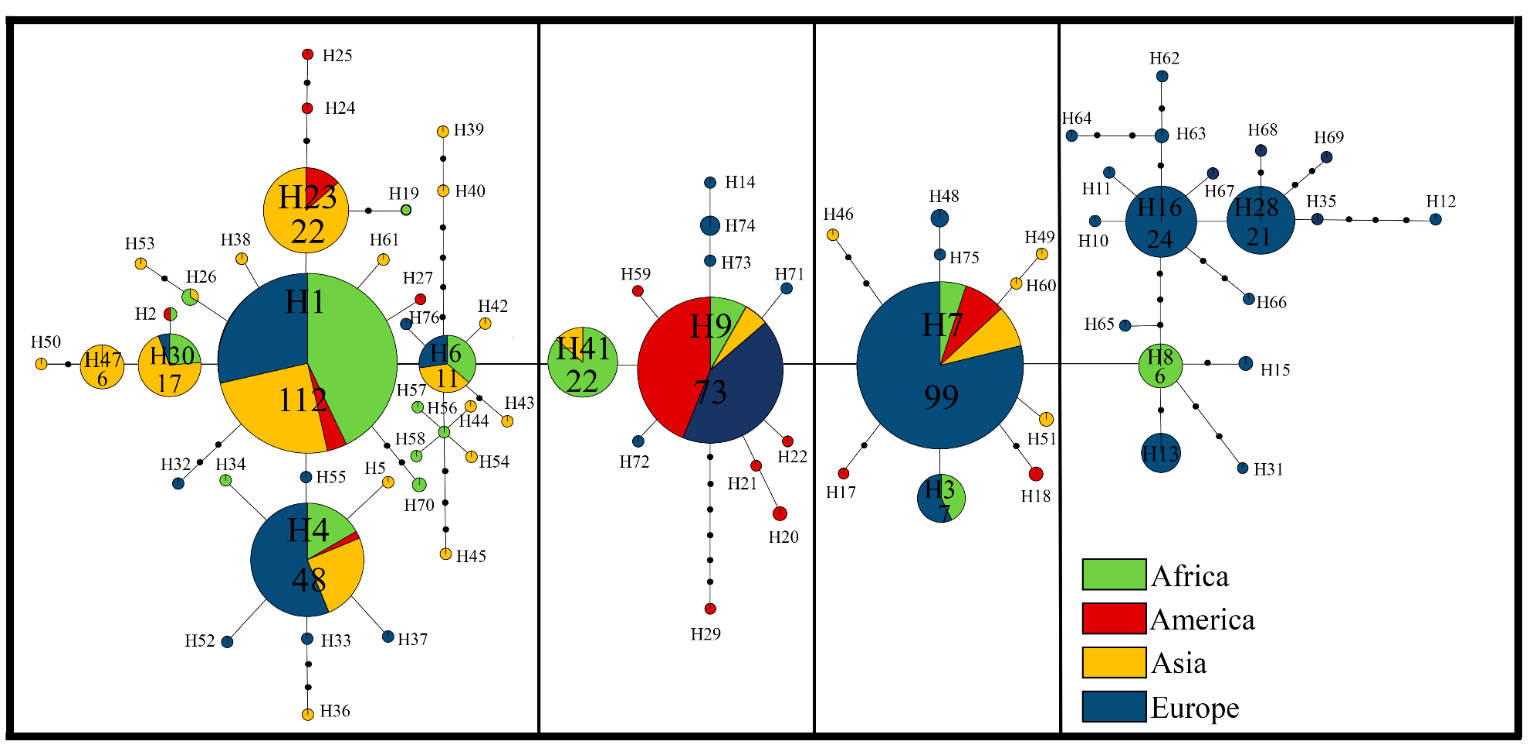


**Figure S1.** Statistical parsimony network of 76 rRNA 18S haplotypes found in 46 populations of *H. canis* in the world. Black dots represent unsampled haplotypes. The frequency of each haplotype is represented by the size of the circle. Each haplotype is coded with a different color according to the continent. The numbers inside the haplotypes in the network indicate the number of individuals that share that haplotype. The four rectangles represent the four haplogroups reported in this study.


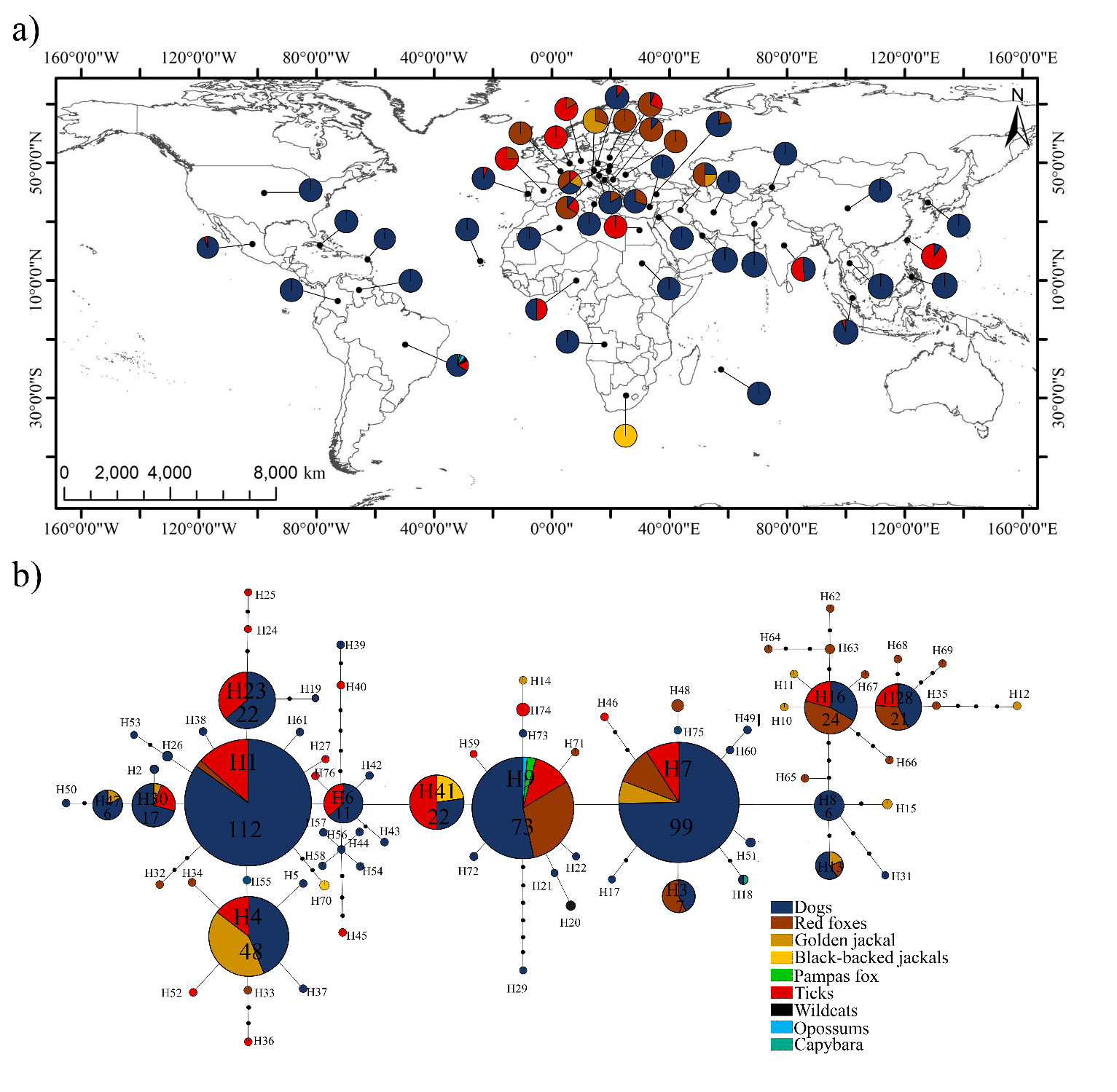


**Figure S2.** Geographical distribution of host and statistical parsimony network of 76 rRNA 18S haplotypes found of *H. canis* in the world. ***a).*** Distribution map of the hosts. Black dots represent sampled populations and pie charts represent hosts found in each sampling population. Section size of pie charts corresponds to the proportion of individuals with given hosts. ***b).*** Haplotype network. Black dots represent unsampled haplotypes. The frequency of each haplotype is represented by the size of the circle. Each haplotype is coded with a different color according to the hosts. The numbers inside the haplotypes in the network indicate the number of individuals that share that haplotype.
